# Impact of caloric and dietary restriction regimens on markers of health and longevity in humans and animals: a summary of available findings

**DOI:** 10.1186/1475-2891-10-107

**Published:** 2011-10-07

**Authors:** John F Trepanowski, Robert E Canale, Kate E Marshall, Mohammad M Kabir, Richard J Bloomer

**Affiliations:** 1 Cardiorespiratory/Metabolic Laboratory, The University of Memphis, Memphis, TN 38152, USA

**Keywords:** caloric restriction, dietary modification, oxidative stress, exercise

## Abstract

Considerable interest has been shown in the ability of caloric restriction (CR) to improve multiple parameters of health and to extend lifespan. CR is the reduction of caloric intake - typically by 20 - 40% of *ad libitum *consumption - while maintaining adequate nutrient intake. Several alternatives to CR exist. CR combined with exercise (CE) consists of both decreased caloric intake and increased caloric expenditure. Alternate-day fasting (ADF) consists of two interchanging days; one day, subjects may consume food *ad libitum *(sometimes equaling twice the normal intake); on the other day, food is reduced or withheld altogether. Dietary restriction (DR) - restriction of one or more components of intake (typically macronutrients) with minimal to no reduction in total caloric intake - is another alternative to CR. Many religions incorporate one or more forms of food restriction. The following religious fasting periods are featured in this review: 1) Islamic Ramadan; 2) the three principal fasting periods of Greek Orthodox Christianity (Nativity, Lent, and the Assumption); and 3) the Biblical-based Daniel Fast. This review provides a summary of the current state of knowledge related to CR and DR. A specific section is provided that illustrates related work pertaining to religious forms of food restriction. Where available, studies involving both humans and animals are presented. The review includes suggestions for future research pertaining to the topics of discussion.

## Introduction

Since the seminal work of McCay et al. [1], much interest has been shown in caloric restriction's ability to improve health and to extend lifespan. Caloric restriction (CR) is the reduction of caloric intake - typically by 20 - 40% of *ad libitum *consumption - while maintaining adequate nutrient intake [2]. In species as diverse as fruit flies [3], guppies [4], and dogs [5], CR has been shown to increase longevity. Also, CR reduces the morbidity of a host of diseases, including (but not limited to), autoimmune diseases, atherosclerosis, cardiomyopathies, cancer, diabetes, renal diseases, neurodegenerative diseases, and respiratory diseases [6,7]. Multiple metabolic pathways have been proposed to be involved in the health-promoting effects of CR, as described in detail previously [8-11]. In addition to an actual reduction in kcal intake, selected nutrients (e.g., resveratrol) [12,13] and drugs (e.g., rapamycin) [14] proposed to mimic the longevity producing effects of CR, are actively being investigated as an alternative to restricting dietary energy.

An alternative to CR, alternate-day fasting (ADF) consists of two interchanging days; one day, subjects may consume food *ad libitum *(sometimes equaling twice the normal intake); on the other day, food is reduced or withheld altogether [15]. Interestingly, while CR regimens typically reduce body weight, ADF regimens often allow for the maintenance of normal body weight, because the subjects may gorge themselves during their feeding days [16]. However, the life-extending benefits of ADF may rival those of CR, particularly in regard to reducing the respective risks of developing type 2 diabetes and cardiovascular disease [15].

While CR reduces caloric intake, CR combined with exercise (CE) both reduces caloric intake (albeit to a lesser extent than CR-only protocols) and increases caloric expenditure. Within the last decade, considerable research has examined whether including exercise in a CR regimen augments the benefits elicited by CR or promotes additional benefits not observed in a CR-only regimen. The results of these studies have been mixed. Depending on the outcome measured, CE either augments a benefit elicited by CR alone [17-20], fails to do so [21-24], or elicits a benefit that was not elicited by CR alone [25].

Dietary restriction (DR) - restriction of one or more components of intake (typically macronutrients) with minimal to no reduction in total caloric intake - is another alternative to CR. While research suggests that neither carbohydrate restriction nor lipid restriction extend life [26-32], protein restriction increases maximum lifespan by roughly 20% [30]. This extension of life may be solely due to the reduction of the amino acid methionine [33].

Many religions incorporate fasting for both spiritual and physical benefits [34,35]. During the holy month of Ramadan, which varies according to the lunar calendar, Muslims abstain from eating or drinking from sunrise (Sahur) to sunset (Iftar). Greek Orthodox Christians fast for a total of 180 - 200 days annually including the following main fasting periods: the Nativity Fast (40 days prior to Christmas), Lent (48 days prior to Easter), and the Assumption (15 days in August). The Biblical-based Daniel Fast typically incorporates a 21-day fasting period in which individuals refrain from consuming animal products, refined carbohydrates, food additives, preservatives, sweeteners, flavorings, caffeine, and alcohol. These above mentioned forms of fasting have been studied using a laboratory-based approach, with findings published in the scientific literature. Although limited objective data are available for other forms of religiously motivated fasts, including but not limited to practices observed in China, Tibet, and India, as well as those of Buddhist monks, it should be noted that fasting within these populations is commonplace. Hence, research investigating the health-specific effects of fasting within these samples is warranted.

The outline of this literature review is as follows: First, data related to CR in both animals and humans will be presented. Next, CE will be reviewed with regard to data obtained from both animal and human investigations. Data will then be presented in relation to ADF in both animals and humans. DR, in particular protein restriction and methionine restriction, will be considered as an alternative to CR. Finally, the three aforementioned forms of religious fasting will be presented. The paper concludes by providing a summary and suggestions for future research.

## Caloric Restriction and Animals

Rodents and primates are the two most commonly studied species in CR animal trials. Many studies use rodents that have been altered genetically to develop various morbidities, including cancer and diabetes. Regarding primates, the majority of work is conducted on rhesus monkeys [36-41].

Typically, caloric intake is reduced by 20 - 40% when compared to *ad libitum*-fed control animals [2,42]. Despite having their caloric intake reduced, the animals are provided sufficient amounts of nutrients and vitamins so as to avoid malnutrition [42]. The magnitude of CR (i.e. the amount of calories that are reduced) is positively correlated with longevity until the point that CR induces starvation [43].

Most studies initiate CR during the weaning phase and continue throughout the animal's lifespan. Short-term CR has also been examined, with some studies lasting as little as 10 days [44]. Similar to magnitude, the duration of CR is positively correlated with longevity [43].

Several animal trials have found that CR improves cardiovascular health [36,39,45-47]. Specifically, CR reduces levels of triglyceride [36,39], phospholipid [36], and total and low density lipoprotein (LDL) cholesterol [39]. CR also increases HDL2b levels [39] and reduces inflammatory markers [36,44,48] such as TNF-α, IL-6, C-reactive protein, and NF-κB. In addition to positive changes in blood lipids, which appear to be associated with reduced risk for cardiovascular disease, several studies have noted significant declines in both blood pressure and heart rate [36,39,45-47]. Moreover, other work has noted cardioprotective alterations in gene expression [45,47].

In addition to cardiovascular-specific effects, CR causes a variety of improvements related to overall health. CR appears to improve glucoregulatory function and insulin sensitivity, particularly in rodents and rhesus monkeys [38,39,48-50]. Specifically, several studies have observed a decrease in fasting blood glucose and insulin [38,39,49,50]. Regarding biomarkers of overall health, reductions in markers of oxidative stress such as hydrogen peroxide, protein carbonyls, and nitrotyrosine have been noted [41,51-53]. Also, CR has been shown to lower brain-reactive antibodies [54] and to reduce T-lymphocyte proliferation [55]. Moreover, CR has been reported to reduce tumor growth [55,56], decrease body weight [39,55-57], reduce sarcopenia [58], maintain neural/cognitive function [57], and improve immune function [54,55]. Collectively, these changes have been associated with an increase in lifespan in many studies involving animals [1,55,59,60].

## Caloric Restriction and Humans

It is difficult to definitively answer whether or not CR prolongs human life because of the ethical and logistical limitations of research design. Rather than measuring longevity directly, most human CR studies measure biomarkers correlated with longevity. Collectively, these studies have noted favorable changes in multifarious biomarkers, particularly those related to cardiovascular and glucoregulatory function.

As noted above, most animal CR studies initiate the restriction of calories during the weaning phase and continue throughout the subject's lifespan. Obviously, no human study has ever initiated CR that early or for that long a duration. Instead, CR is typically instituted for a period of 6 - 12 months [61-71]. However, a few studies have examined CR for 2 years [72-74], ~6 years [75-77], and longer [78].

The majority of work has been conducted on healthy, middle aged, non-obese (normal or overweight) men and women, with a significant amount of investigations utilizing data obtained from the CALERIE (Comprehensive Assessment of Long Term Effects of Reducing Caloric Intake) program [61,64-67,69-71]. This program was initiated by the National Institute on Aging to investigate the adaptive responses of CR on free-living humans [79]. In addition to the CALERIE program, another investigation of importance, Biosphere 2, contained eight subjects aged from 27-67 years [72-74]. These subjects lived in an enclosed ~3 acre "ecological mini-world" for two years [79]. Food intake was subnormal due to the inability to grow enough food for *ad libitum *consumption; hence, subjects followed a CR plan by default. Aside from normal weight men and women, investigations involving CR have also included obese [80-83], diabetic [83], young [84,85], and elderly [78,86] individuals.

Somewhat similar to the animal studies, daily caloric intake has usually been reduced by 20-25% of the caloric intake of *ad libitum*-fed control subjects [61-71,84]. Alternatively, some studies have reduced caloric intake by a fixed number of calories rather than a percentage of usual intake [82,83].

As mentioned above, numerous studies have found that CR improves cardiovascular and glucoregulatory health. Specifically, CR may reduce the risk of cardiovascular disease by lowering total cholesterol, triglycerides, blood pressure, and carotid intima-media thickness [62,67,72,75,77]. CR also has been shown to attenuate the age-related decline in diastolic function [77]. Regarding glucoregulatory health, circulating insulin [64,70,73-75,86] and glucose [70,73-75] levels decrease - while insulin sensitivity increases [70] - following a period of CR.

CR has also been shown to attenuate oxidative stress [61,65,80-84,87], a condition thought to contribute to aging and disease [88]. In addition, enhanced verbal memory performance has been reported in elderly individuals on a CR regimen as assessed by the Rey Auditory Verbal Learning Task [86]. Unfortunately, CR does not appear to retard the age-related loss of bone [69] and muscle [71] mass.

## Caloric Restriction combined with Exercise

### Animals

A number of studies have examined whether the addition of exercise to a CR regimen (CE) augments any of the health-promoting benefits caused by CR alone. Animal CE studies often feature a minimum of four groups: 1) a sedentary group fed *ad libitum*, 2) a sedentary group fed a CR diet, 3) a voluntary exercise group fed *ad libitum*, and 4) a voluntary exercise group fed a CR diet. Male rats are often the animal of choice for CE studies, because they do not increase their caloric intake to compensate for their exercise-induced caloric expenditure [22]. Voluntary wheel running is typically the exercise of choice for these studies.

Some studies have found that CE does not elicit health-promoting benefits beyond those elicited by CR [21-24]. The presence or absence of exercise does not appear to affect oxidative stress levels or pro-inflammatory protein levels in animals fed an 8% CR diet [21,24]. However, CE reduces C-reactive protein levels to a greater extent than CR by itself [19]. Regarding myocardial health, CE lowers the likelihood of developing both myocardial necrosis and myocardial ischemia [17,18]. CE has been shown to attenuate age-related sarcopenia to a greater extent than CR by itself [20]. Moreover, CE reduces muscle fatigue and may increase the oxidative capacity of muscle fibers [25]. However, the addition of exercise to a CR regimen does not appear to affect an animal's maximal life span, the outcome of most interest to most investigators [22]. Future work in this area should focus on how different exercise intensities, volumes, frequencies, and modalities affect the ability of exercise to augment the health-promoting effects of CR.

### Humans

Several CE studies have involved human subjects [61,64,66,67,84,89-91]. Unlike the majority of the animal CE studies, human CE studies typically quantify the caloric expenditure caused by the exercise regimen. Many human CE studies have incorporated a total caloric reduction of 25%, with 12.5% coming from exercise-induced expenditure and another 12.5% coming from reduced caloric intake (i.e. diet) [61,64,66,67,89]. The expenditure is often accomplished by performing aerobic exercise several (e.g. 5) days per week [61,64,66,67,89].

Many investigations have noted no significant difference between CE and CR regarding their respective effects on fasting insulin levels [64], DNA damage [61], muscle mitochondrial gene expression [61], triglyceride levels [67], and liver lipid content [66]. In contrast to these findings, two investigations have noted a further reduction in both diastolic blood pressure and LDL cholesterol with CE when compared to CR alone [67,89]. Moreover, other work has noted that CE improved insulin sensitivity, while CR-alone failed to do so [89]. Also, CE has been shown to increase bone mineral density at the femoral neck and reduce sTNFR1, an inflammatory biomarker, in overweight postmenopausal women [90]. Collectively, although results are somewhat mixed, it appears that the addition of exercise to a CR plan may provide further health benefits. Perhaps of main importance, it may be easier for an individual to comply with a CE regimen than a CR regimen when total caloric reduction (increased caloric expenditure combined with decreased caloric intake) is held constant. For example, a 12.5% reduction in dietary energy coupled with a 12.5% increase in expenditure due to exercise appears much more manageable than a 25% reduction in dietary energy (as is the case in many of the CR-only plans). In an attempt to determine the optimal plan for improved health, future work may investigate varying amounts of exercise-induced caloric expenditure (i.e. less than or greater than 12.5%) coupled with varying amounts of decreased caloric intake. Indeed, compliance is of major importance when considering long-term CR regimens.

## Alternate-Day Fasting and Animals

ADF consists of alternating 24-hour periods of *ad libitum *intake ("feast period") and partial or complete restriction of caloric consumption ("fast period"). Some studies have withheld food altogether during the fast period, while others have restricted caloric consumption by as little as 25% of *ad libitum *intake [92]. Unlike CR, ADF need not necessarily reduce overall caloric consumption or bodyweight, because subjects may compensate for the reduced caloric intake during fast periods by gorging themselves during feast periods [15,16].

ADF has been found to extend lifespan in several animal trials [16,93,94]. Interestingly, Duan and colleagues suggest that an increase in brain-derived neurotrophic factor (BDNF) may mediate the observed life extension caused by an ADF regimen [94]. The ability of ADF to retard or prevent altogether the development of many morbidities, including cardiovascular disease, kidney disease, cancers, and diabetes, may also explain some of the observed increases in longevity [15,16,94-99].

Many animal ADF studies have noted improvements in cardiovascular function. Both resting heart rate and blood pressure are reduced following a period of ADF [96,98,99]. Heart rate variability, which is associated with improved cardiovascular function and a reduced probability of heart failure, has been noted to be favorably affected by ADF in rats [100]. Ahmet and colleagues [95] noted a prophylactic ability of ADF to attenuate the development of post-infarct chronic heart failure. In this work, the investigators induced myocardial infarction (MI) in Sprague-Dawley rats that had been following a diet of either ADF or *ad libitum *intake. When compared to the *ad libitum*-fed group, the rats on the ADF schedule had less left ventricle remodeling and functional decline following the MI.

Glucoregulatory function is also typically improved following an ADF regimen. ADF can improve insulin sensitivity, which results in lower fasting glucose and insulin concentrations and improved glucose tolerance [16,99]. However, Mager et al. [96] noted that glucose concentrations did not change in Sprague-Dawley rats following a period of ADF, although glucose concentrations were reduced following a 40% CR plan during the same time period. In contrast, Anson et al. [16] noted that glucose and insulin concentrations were reduced by a similar extent (compared to a control group) in both an ADF group and a 40% CR group.

One of the most germane questions regarding ADF is whether or not it can elicit benefits that are comparable to CR, a question best answered by including both plans within the same research design. While some studies have done just that, as indicated by the work of Anson et al. [16] and Mager et al. [96] presented above, most have not. This highlights one of the main directions that should be taken in future research regarding CR and ADF.

## Alternate-Day Fasting and Humans

Of the ADF trials that have been performed, relatively few have used human subjects. Originally, human trials were performed simply to examine the feasibility of maintaining an ADF regimen [101]. Now that feasibility has been established, research turns to examining what health-related benefits ADF can yield and through which mechanisms these benefits are yielded.

In human ADF trials, subjects have been permitted to consume anywhere from 0% to 50% of the estimated daily energy required to maintain body mass during fast periods [102]. Few human ADF studies incorporate a fasting period lasting longer than 20 weeks due to ethical and logistical (i.e. compliance) limitations. In fact, many trials have lasted only a few days. Most subjects are able to remain compliant throughout their fast, and few experience any fast-related health complications. However, as expected, subjects often report hunger and irritability during fast days (as measured via questionnaires), which calls into question the sustainability of long-term ADF diets [101]. Despite being able to consume food *ad libitum *during feast days, human subjects sometimes experience weight loss as a result of the ADF regimen. By contrast, animals often maintain bodyweight by gorging themselves during feast periods [15,16]. Interestingly, Heilbronn and colleagues [101] examined nonobese humans and noted that subjects that classified themselves as "big eaters" lost less weight than those that claimed that they "watched what they ate." This suggests the possibility that obese subjects might gorge themselves during feast days and consequently not lose weight on an ADF regimen.

Human trials have noted mixed findings with ADF regarding glucoregulatory function. Heilbronn and colleagues [101] noted a decrease in fasting insulin but no difference in fasting glucose following 22 days of ADF. Another study by the same group [103] found that women on a 22 day ADF regimen cleared serum glucose following a test meal (500 kcal, 12.2 g fat, 90 g carbohydrate, 17.6 protein) less efficiently when compared to pre-fast values; no difference in glucose clearance efficiency was seen in men. In the same study, men on an ADF diet experienced a reduced insulin response to the test meal, but this effect was not observed in women. Taken together, these findings suggest that men and women may respond differently to ADF. In addition, Halberg et al. [104] found no change in fasting glucose or insulin concentrations in men (this study did not include women) following a 14-day ADF program. Future studies should examine potential causes for the sex-specific differences noted above.

Johnson and colleagues [105] noted substantial benefits from an 8-week long ADF regimen on overweight, asthmatic subjects. Nine subjects consumed food *ad libitum *on feast days and one meal replacement shake, which consisted of either 320 or 380 kcals for women and men, respectively, on fast days. Peak Expiratory Flow (PEF) significantly increased within 2 weeks of the ADF diet; however, forced expiratory volume (FEV_1_) did not increase following the protocol. The beneficial effects of albuterol administration were greater following the 8 weeks of ADF compared to baseline values. The authors suggest that the diet improved "bronchial responsiveness." Scores on questionnaires such as the Juniper mini-Asthma Quality of Life Questionnaire (mini-AQLQ) and Asthma Symptom Utility Index (ASUI) indicated that subjects' quality of life was improved following the ADF regimen.

Johnson and colleagues have examined ADF regimens over the course of several years, including over 500 subjects as of 2006. The authors have noted improvements in the following: insulin sensitivity, asthma, seasonal allergies, autoimmune diseases such as rheumatoid arthritis, osteoarthritis, infectious disease of viral, bacterial, and fungal origin, inflammatory central nervous system lesions involved with Tourette's syndrome and Meniere's disease, cardiac arrhythmias, and menopause-related hot flashes [102]. Much remains to be known regarding the mechanisms responsible for the effects of ADF on these outcomes. Moreover, data pertaining to the use of ADF in human subjects are not yet available in regard to biomarkers specific to blood lipids, oxidative stress, and inflammation. Clearly, this area of research has the potential for new discovery.

## Dietary Restriction

Whereas CR is a reduction of caloric intake, DR is a specific reduction/manipulation in nutrient intake. DR need not necessarily result in CR, because the reduced intake of a particular nutrient (typically a macronutrient) may be offset by an increased intake of one or more other nutrients. For example, if carbohydrate intake is reduced, then protein and/or lipid intake can be increased so as to maintain normal caloric intake. Given this, DR appears to be considerably more tolerable than CR, especially when considering that some researchers estimate that energy consumption must be reduced by a minimum of 20 - 25% in order to beget any life-extending benefits from a CR regimen [59,106,107]. Consequently, many studies have attempted to determine if DR can elicit life-extending effects comparable to CR. This area of study may be partly motivated by the work of Simpson and coworkers [108] who have proposed that state-space geometric models can be used to determine the influence of kcal reduction and specific nutrients on longevity and associated outcome measures.

Neither carbohydrate restriction nor lipid restriction appear to be effective alternatives to CR. Lipid restriction has been shown to have no effect on longevity [26,27]. Regarding carbohydrate, several studies have found that increasing intake either increases or has no effect on longevity, suggesting that restriction would not extend life [28,29], also reviewed in [30]. Moreover, both forms of macronutrient restriction fail to decrease reactive oxygen species production or oxidative DNA damage [31,32].

Protein restriction appears to be a viable candidate for an alternative to CR. Sixteen out of 18 reviewed experiments found that protein restriction increased maximum lifespan in rodents (reviewed in [30]). The average increase in maximum lifespan in the 16 positive studies was approximately 20%. When this is compared to the ~40% increase in lifespan found in many CR investigations, it suggests that protein restriction accounts for approximately half of this effect [30]. Moreover, several of the reviewed studies compensated for the reduction in protein by increasing carbohydrate intake; this balanced total caloric intake and ensured that the prolongation of life was due to restriction of protein and not calories.

In regard to the above, several studies have examined whether restriction of an individual amino acid accounts for most or all of the life-extending benefits of protein restriction. While a few studies have found that tryptophan restriction increases lifespan [109,110], the majority of studies have focused on methionine restriction. As with tryptophan restriction, methionine restriction increases longevity [111-115]. Forty percent methionine restriction has been reported to decrease both mitochondrial reactive oxygen species generation and oxidative damage in mitochondrial DNA [116,117]. Further evidence that methionine restriction increases longevity (reviewed in [118]): 1) methionine content has an inverse relationship with maximum life span in mammals [119]; 2) of the amino acids, methionine is one of the most vulnerable to oxidation by reactive oxygen species [120]; 3) methionine supplementation increases LDL cholesterol oxidation [121]; and 4) raising methionine intake increases plasma homocysteine concentrations, which in turn elevates the risk of cardiovascular disease and mortality [121].

In an effort to disentangle the respective effects of methionine restriction and protein restriction, Caro and coworkers [33] examined the impact of 40% restriction of all dietary amino acids except methionine on biomarkers of aging. The investigators found that this restriction failed to reduce both mitochondrial reactive oxygen species generation and oxidative damage in mitochondrial DNA. The authors concluded that methionine is the only amino acid that can affect mitochondrial reactive oxygen species generation and subsequent oxidative stress by manipulating its intake.

In summary, neither carbohydrate restriction nor lipid restriction appear to be responsible for the life extension caused by CR, while approximately half of the life extension effect of CR seems to be ascribable to protein restriction. A wealth of evidence indicates that methionine restriction might account for most or all of the life-extending benefits of protein restriction. Fortunately, a methionine-restricted diet is both feasible and tolerable [122], suggesting that it might be an attractive alternative to CR for those seeking the health-enhancing properties of such a plan. However, because the evidence provided above has been generated using animal models, further work involving human test subjects is necessary before firm conclusions can be made.

## Religious Fasting

Several religions place one or more of the following restrictions on food consumption: 1) the types of foods permitted for consumption in general or during particular times of the year; 2) the time of day when food consumption is permitted; and 3) food preparation [34]. These types of restrictions can either persist year-round or be active only during special fasting periods. The text below focuses on the following fasting periods: 1) Islamic Ramadan; 2) the three principal fasting periods of Greek Orthodox Christianity (Nativity, Lent, and the Assumption); and 3) the Biblical-based Daniel Fast. The reason for the inclusion of these religious fasts in this review and the exclusion of others is that, to our collective knowledge, these are the only fasts about which scholarly research has been performed that explicitly detailed the subjects' dietary intake.

### Ramadan

During the holy month of Ramadan, all healthy adult Muslims are forbidden from consuming any food or water from sunrise (Sahur) to sunset (Iftar). Food and fluid intake become nocturnal during Ramadan, and the common practice is to eat one large meal after sunset and one lighter meal before dawn [123]. Ramadan is clearly the most commonly researched religious fast. The collective work highlights both the positive and negative effects of Ramadan fasting on overall health.

It was previously thought that Ramadan fasting often led to reduced energy intake and weight loss [124], but recent studies have found that caloric intake actually increases despite the decreased meal frequency [125]. In relation to macronutrient composition, meals are often composed of more fat and less carbohydrate during Ramadan than during the rest of the year [126,127].

Ramadan fasting has elicited mixed results in relation to cardiovascular health, particularly regarding lipid profiles. Hallak and Nomani [124] and Ziaee et al. [128] noted a decline in high density lipoprotein (HDL)-C levels and an increase in LDL-C levels following Ramadan fasting. However, several other studies have found an increase in HDL-C levels and a decline or no change in LDL-C levels [125,127,129-131]. Indeed, some studies have noted an increase in HDL-C levels by as much as 20% [125] and 30% [131]. The majority of studies have found no difference in triglyceride levels [130-132], while one study noted a decrease following a period of Ramadan fasting [123]. Salehi and Neghab [132] reported declining total cholesterol levels following a Ramadan fast. Conversely, Aksungar et al [130] did not note any changes in total cholesterol and LDL-C levels but did note decreases in the TC/HDL ratio, C-reactive protein levels, and homocysteine levels.

Changes in heart rate and blood pressure have also been observed during Ramadan. Husain et al [133] observed a significant lowering of resting heart rate in men, although no changes were noted regarding the resting heart rate of women. Heart rate during steady-state aerobic exercise has also been shown to decrease during the fast compared to non-fasting values [134,135]. Regarding blood pressure, one study noted a decrease in both resting systolic and diastolic blood pressure -although this change was noted in both fasters and non-fasters, raising a question regarding the ability of Ramadan fasting to actually promote such an effect [136] while four studies found no difference in either variable [135,137-139]. Ramadan (2002) [135] compared blood pressure changes during steady-state aerobic exercise during and after Ramadan fasting. Systolic - but not diastolic - blood pressure significantly increased during exercise when subjects were fasting. Contrastingly, neither systolic nor diastolic blood pressure changed during exercise in subjects who were not fasting.

To our knowledge, only two studies to date have examined the effects of Ramadan fasting on oxidative stress and antioxidant status, highlighting an important area for future research. Ibrahim et al [123] observed a reduction in malondialdehyde (MDA) erythrocyte concentrations, while no changes were noted regarding levels of either serum MDA or plasma protein-bound carbonyls. No changes were found regarding the concentration of glutathione or the activities of glutathione peroxidase and catalase in erythrocytes. Plasma levels of ß-cryptoxanthin and total carotenoids significantly decreased during Ramadan fasting, and plasma levels of vitamin C, β-carotene, lycopene, and lutein were non-significantly reduced. No changes were noted regarding plasma levels of α-tocopherol, γ-tocopherol, retinol, α-carotene, and zeaxanthin. Chaouachi and colleagues [140] reported that blood levels of vitamin A increased, while blood levels of vitamin E decreased during Ramadan fasting.

As with the reports of Ramadan's effects on markers of cardiovascular health, similarly mixed results are available regarding Ramadan fasting and the ability to improve glucoregulatory health. Two studies have noted a decrease in fasting blood glucose following Ramadan [123,132], while a recent study noted no effect on blood glucose levels [131]. Kassab et al [141] noted that serum leptin levels increased during Ramadan by 37% and 39% in obese and nonobese subjects, respectively. Additionally, the authors found a significant correlation between changes in serum leptin and serum insulin levels, which suggests that insulin may play a role in regulating leptin secretion [141].

Aside from the potentially beneficial effects discussed above, some studies have noted adverse effects associated with Ramadan fasting. Ramadan fasting has been shown to negatively impact nocturnal sleep by increasing sleep latency and decreasing both slow wave sleep and rapid eye movement sleep [142,143]. Lack of sleep can cause irritability [144] and has been shown to lead to an increase in the intake of stimulants such as coffee and tea during the month of Ramadan [145]. Ramadan fasting may lead to ulcer complications, as studies have reported increased gastric acidity during the day, with altered circadian patterns of levels of plasma gastrin, gastric pH, glucose and calcium [146]. Other potential adverse health effects associated with Ramadan fasting include energy level imbalances [124], dehydration [147], decreased athletic performance [148], and altered circadian fluctuations in hormone levels [149].

Collectively, the above-listed studies have reported conflicting effects of Ramadan fasting on a number of health-related biomarkers. There are several potential confounding variables that may influence the effect of Ramadan fasting on these biomarkers, including: age, physical activity, diet, sleep cycles, and cultural habits [126,150]. Regarding the latter, El-Ati et al. [126] reported that total energy intake increased among Saudi subjects and decreased among Indian subjects during Ramadan, and that this discrepancy was due to the differences in food choices between the two groups. Food choices and eating habits affect metabolism and could potentially affect the above-listed biomarkers. Future investigations should take these variables - as well as other confounding variables - into consideration.

### Greek Orthodox

There are three principal fasting periods for Greek Orthodox Christians. During the 40 days that precede Christmas (Nativity), dairy products, eggs, and meat are proscribed every day, while fish and olive oil are also forbidden on Wednesdays and Fridays. During the 48 days that precede Easter (Lent), dairy products, eggs, and meat are proscribed. Olive oil consumption is permitted only on weekends during this period, and fish consumption is only allowed on March 25^th ^and Palm Sunday. During the first 15 days of August (the Assumption), dairy products, eggs, and meat are proscribed. Olive oil consumption is permitted only on weekends during this period, and fish consumption is only allowed on August 6^th^. Cheese, eggs, fish, meat, milk, and olive oil are also proscribed on every Wednesday and Friday that falls outside of the principal fasting periods. This latter proscription is temporarily lifted on the week following Christmas, Easter, and the Pentecost. Collectively, dietary consumption is restricted for 180-200 days each year.

Most studies have reported a decreased caloric intake during the fasting periods [151-154], which may result in lowered body mass [151,152]. Percentagewise, carbohydrate intake appears to increase, while both protein and fat intake decrease [151-153]. Both saturated fat and trans-fatty acid consumption appear to decrease during fasting periods, while both monounsaturated and polyunsaturated fat consumption do not change [151,153].

Both total cholesterol and LDL-C levels decrease during fasting periods [151,152], while conflicting results exist regarding HDL-C levels [151,152]. One study noted that the LDL/HDL ratio does not change during Greek Orthodox Christian fasting [152], while conflicting results have been noted regarding both the total/HDL ratio and triglyceride levels [151,152]. Greek Orthodox Christian fasting appears to have no effect on blood glucose levels [151,152], although fiber intake increases during fasting periods [151-155].

Both riboflavin [151] and calcium [151,153,155] intake appear to decrease during fasting periods, while magnesium intake appears to increase [151,155]. The intake of the following vitamins and minerals do not appear to change during fasting periods: vitamin A [151,153]; thiamin [151]; niacin [153]; vitamin B_12 _[151,153]; vitamin C [151,153,154]; vitamin E [151,153]; phosphorus [151]; potassium [151,155]; and zinc [151]. Mixed results have been recorded regarding the intake of both folate [151,153,155] and sodium [151,155].

To our knowledge, only one study has examined the effects of Greek Orthodox Christian fasting on other hematological variables [154]. The authors reported that fasters experienced a relative increase in serum ferritin levels, a relative decrease in MCHC levels, and no relative change in levels of hemoglobin, serum iron, and transferrin when compared with non-fasters during the Christmas fasting period. The authors also reported that the non-fasters' hematocrit declined significantly (42.7 ± 5.1 vs. 41.2 ± 5.1%) when compared to the changes in fasters' hematocrit (41.2 ± 3.9 vs. 40.0 ± 5.3%). However, this change is of little to no clinical relevance, as all values were within normal range.

There are conflicting findings on the effects of Greek Orthodox Christian fasting on blood pressure. One study found that systolic blood pressure increased during fasting periods [151], while another study found no change in systolic blood pressure when fasters were compared with non-fasters [155]. One study reported that non-fasters' diastolic blood pressure decreased significantly during fasting periods when compared to the changes in fasters' diastolic blood pressure [155], while another study reported that fasters' diastolic blood pressure did not change during fasting periods [151].

In summary, Greek Orthodox Christian fasting appears to lower caloric intake and body mass, and both total and LDL-C decrease during fasting periods. The intake of most vitamins and minerals do not appear to change during these periods, although riboflavin and calcium intake each appear to decrease, and magnesium intake appears to increase. More research remains to be performed on hematological variables and blood pressure during fasting periods due to both the lack of previous research and the inconclusive findings. Also, future studies should examine each of the three principal fasting periods both separately and aggregately, because each fasting period has unique food proscriptions and durations.

### Daniel Fast

A popular fast practiced by many Christians and Jews is the Daniel Fast, based on the Biblical story of Daniel (1:8-14), in which Daniel resolved not to defile himself with the royal food and wine, and he asked the chief official for permission to provide to him and his three friends nothing but vegetables to eat and water to drink for 10 days. Later in the same book (Daniel 10:2-3), Daniel again partook in a 21 day period of "clean" eating, during which time he ate no choice food (meat or wine). Based on these two stories, a modern day Daniel Fast involves *ad libitum *intake of certain foods, but the food choices are restricted to essentially fruits, vegetables, whole grains, nuts, seeds, and oil. In essence, this plan is a form of DR and resembles a vegan diet, which has been reported to yield health-enhancing properties [156,157]. However, a Daniel Fast is much more stringent, because preservatives, additives, sweeteners, flavorings, caffeine, and alcohol are each forbidden. However, because individuals traditionally follow this fast for religious purposes in an attempt to become "closer to God" during a time of extended prayer, the anecdotal reports have indicated excellent compliance.

To test the health benefits of the Daniel Fast within a laboratory-based protocol, we recently enrolled 43 subjects (13 men; 30 women; 35 ± 1 yrs; range: 20-62 yrs) to complete a 21-day Daniel Fast. Pre and post intervention, subjects underwent a variety of tests including measures of body weight and composition (measured via dual energy x-ray absorptiometry), resting blood pressure and heart rate, fasting blood measures of oxidative stress, inflammation, blood lipids, insulin, and glucose. Subjects' self reported compliance, mood, and satiety in relation to the fast were also recorded.

We noted excellent compliance to the fast (98.7 ± 0.2%; mean ± SEM), as well as excellent mood and satiety (7.9 ± 0.2 using a 10 point scale). The following variables related to cardiovascular disease risk were significantly (p < 0.05) lower following the fast as compared to before the fast: total cholesterol, LDL-C, SBP, and DBP. Insulin, HOMA-IR, and C-reactive protein, although lowered to a clinically meaningful extent, were not of statistical significance. Unfortunately, due to the drastic decrease in total cholesterol, HDL-C was lower after the fast as compared to before the fast (55.65 ± 2.50 vs. 47.58 ± 2.19 mg·dL^-1^). Although body weight and body fat were reduced slightly, no significant difference was noted [158]. In reference to measures of oxidative stress, we noted a significant increase (p < 0.05) in Trolox Equivalent Antioxidant Capacity, and a significant decrease (p < 0.05) in malondialdehyde and hydrogen peroxide [159]. Similar results as presented above have been noted in a recent follow-up investigation of the Daniel Fast (unpublished findings).

The above data demonstrate that a Daniel Fast can significantly improve several biomarkers of overall health, particularly those related to cardiovascular and metabolic disease. Larger scale, randomized studies will be needed to extend these preliminary findings. Future studies should consider extending the duration of the fast, as well as modifying food choices in an effort to maintain HDL-C levels.

## Overall Summary and Conclusions

CR has been demonstrated to extend the maximal lifespan of a diverse group of species. This extension of life is maximized when: 1) the magnitude of CR is elevated to the highest possible value before inducing malnutrition and 2) the duration of CR is maximized. Animals on CR regimens exhibit a variety of improvements in overall health in general and cardiovascular health in particular. Unfortunately, the likelihood of discovering whether or not CR extends human life is rather remote due to the ethical and logistical limitations of research design. The optimal magnitude and duration of CR for humans will also likely never be known for the same reason. Nonetheless, many human CR studies have noted favorable changes in biomarkers related to cardiovascular and glucoregulatory function, which likely relate to quality of life and may relate to longevity.

Due to the austerity of following a CR regimen of sufficient magnitude and duration, alternatives such as CE, ADF, and DR may prove to be more appealing. The most pertinent consideration to make when evaluating these alternatives is whether or not they elicit benefits that are comparable to CR. ADF has been demonstrated to extend life and improve both cardiovascular and glucoregulatory function in animals. Human trials have noted heterogeneous findings and sex-specific differences regarding ADF's effects on glucoregulatory function. Unfortunately, it is difficult to compare the effects of ADF and CR regimens across different studies due to an enormous number of confounding variables. Future studies should feature an ADF group and a CR group so that direct comparisons can be made. Regarding DR, neither carbohydrate restriction nor lipid restriction extend life. However, protein restriction appears to extend maximum lifespan by 20%. Recent findings suggest that methionine restriction may be the single cause of life extension observed in protein restriction studies. Future studies should examine the effects of different magnitudes of methionine restriction on life extension.

As noted above, considerable research has recently focused on whether CE augments any of the health-promoting benefits caused by CR alone. Very few conclusions can currently be made due to the mixed results of the studies. CE does not appear to extend life beyond any extension observed to be caused by CR. However, CE may be more manageable for many individuals due to the smaller reduction in dietary calorie consumption. Future work in this area should examine different exercise intensities, volumes, frequencies, and modalities. Also, future work should compare different amounts of exercise-induced caloric expenditure.

Islamic Ramadan, the three principal fasting periods of Greek Orthodox Christianity, and the Daniel Fast each provide a unique and interesting vantage point for evaluating the effects of food restriction/modification. The majority of findings related to Ramadan fasting are mixed, and these discrepancies are most likely due to the differences in cultural norms - particularly dietary norms - of the groups studied. The three Greek Orthodox Christian fasts appear to decrease body mass and lower both total and LDL cholesterol levels, although these fasts minimally affect the intake of most vitamins and minerals. Finally, the Daniel Fast is associated with profound and favorable effects on a variety of markers related to human health, including blood pressure, blood lipids, insulin sensitivity, and biomarkers of oxidative stress.

This paper has touched on some of the numerous methods of restricting dietary intake. Whether one chooses to restrict energy intake daily, fast every other day, restrict intake of a particular macronutrient, or fast for religious purposes, the authors hope that this paper can serve as a valuable tool to understanding the ability of dietary modification to improve overall health and the quality of life. Furthermore, we hope that this information will fuel the development of new ideas and research studies focused on investigating the health benefits of caloric and dietary restriction.

## Abbreviations

ADF: alternate-day fasting; ASUI: Asthma Symptom Utility Index; BDNF: brain-derived neurotrophic factor; CALERIE: Comprehensive Assessment of Long Term Effects of Reducing Caloric Intake; CE: caloric restriction combined with exercise; CR: caloric restriction; DR: dietary restriction; FEV_1_: forced expiratory volume; HDL: high density lipoprotein; LDL: low density lipoprotein; MDA: malondialdehyde; MI: myocardial infarction; mini-AQLQ: mini-Asthma Quality of Life Questionnaire; PEF: Peak Expiratory Flow

## Competing interests

The authors declare that they have no competing interests.

## Authors' contributions

JFT, REC, KEM, MMK, and RJB were all involved in the review of relevant literature pertaining to this topic and in the writing and editing of this manuscript. All authors read and approved the final manuscript.
